# 6-Amino-3-methyl-4-(3-nitro­phen­yl)-1-phenyl-1*H*,4*H*-pyrano[2,3-*c*]pyrazole-5-carbonitrile

**DOI:** 10.1107/S1600536811017387

**Published:** 2011-05-20

**Authors:** Ming-Shu Wu, Du-Lin Kong, Xiang-Zhu Zhang

**Affiliations:** a Department of Chemical Engineering, Anyang Institute of Technology, Anyang, Henan 455000, People’s Republic of China; b College of Chemistry & Chemical Engineering, Hainan Normal University, Haikou 571158, People’s Republic of China

## Abstract

The title compound, C_20_H_15_N_5_O_3_, was synthesized by the one-pot reaction of a four-component reaction protocol in aqueous medium. The pyrano[2,3-*c*]pyrazole system is essentially planar, with a maximum deviation of 0.026 (2) Å. The 3-nitro­phenyl and phenyl rings make dihedral angles of 81.11 (5) and 13.36 (1)°, respectively, with the mean plane of the pyrano[2,3-*c*]pyrazole ring. The crystal structure is stabilized by N—H⋯N hydrogen bonds, which form infinite chain propagating along the *c* axis and by N—H⋯O hydrogen bonds, which form infinite chains propagating along the *a* axis. There are also N—O⋯N—C dipole–dipole inter­actions along the *a* axis with an O⋯N distance of 3.061 (3) Å, which is shorter than that of the N—H⋯O hydrogen bond [3.196 (3) Å].

## Related literature

For the anti­microbial, insecticidal and anti-inflammatory activity of pyran­opyrazole derivatives, see: El-Tamany *et al.* (1999 ▶); Ismail *et al.* (2003 ▶); Zaki *et al.* (2006 ▶) and for their applications as pharmaceutical ingredients and biodegradable agrochemicals, see: Junek & Aigner (1973 ▶); Sharanin *et al.* (1983 ▶); Vasuki & Kumaravel (2008 ▶); Wamhoff *et al.* (1993 ▶). For the Chk1 kinase inhibitor, see: Foloppe *et al.* (2006 ▶). For the use of multi-component reaction (MCR) protocols in water in the development of libraries of medicinal scaffolds, see: Chanda & Fokin (2009 ▶); Tejedor & Garcia-Tellado (2007 ▶).
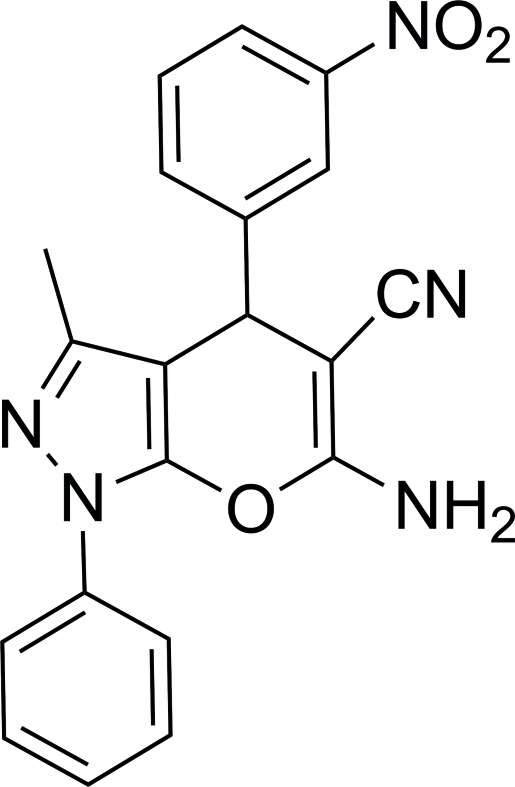


## Experimental

### 

#### Crystal data

C_20_H_15_N_5_O_3_
*M* *_r_* = 373.37
Monoclinic, 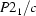

*a* = 9.5089 (8) Å
*b* = 13.9137 (11) Å
*c* = 13.3747 (12) Å
β = 96.263 (1)°
*V* = 1759.0 (3) Å^3^
*Z* = 4
Mo *K*α radiation
μ = 0.10 mm^−1^
*T* = 298 K
0.50 × 0.48 × 0.47 mm

#### Data collection

Bruker SMART CCD area-detector diffractometer
Absorption correction: multi-scan (*SADABS*; Bruker, 2002 ▶) *T*_min_ = 0.952, *T*_max_ = 0.955
8659 measured reflections
3087 independent reflections
1961 reflections with *I* > 2σ(*I*)
*R*_int_ = 0.037

#### Refinement

*R*[*F* ^2^ > 2σ(*F* ^2^)] = 0.042
*wR*(*F* ^2^) = 0.121
*S* = 1.07
3087 reflections
255 parameters
H-atom parameters constrained
Δρ_max_ = 0.19 e Å^−3^
Δρ_min_ = −0.17 e Å^−3^

### 

Data collection: *SMART* (Bruker, 2002 ▶); cell refinement: *SAINT* (Bruker, 2002 ▶); data reduction: *SAINT*; program(s) used to solve structure: *SHELXS97* (Sheldrick, 2008 ▶); program(s) used to refine structure: *SHELXL97* (Sheldrick, 2008 ▶); molecular graphics: *SHELXTL* (Sheldrick, 2008 ▶); software used to prepare material for publication: *SHELXTL*.

## Supplementary Material

Crystal structure: contains datablocks I, global. DOI: 10.1107/S1600536811017387/zk2006sup1.cif

Structure factors: contains datablocks I. DOI: 10.1107/S1600536811017387/zk2006Isup2.hkl

Supplementary material file. DOI: 10.1107/S1600536811017387/zk2006Isup3.cml

Additional supplementary materials:  crystallographic information; 3D view; checkCIF report

## Figures and Tables

**Table 1:** Hydrogen-bond geometry (Å, °)

*D*—H⋯*A*	*D*—H	H⋯*A*	*D*⋯*A*	*D*—H⋯*A*
N3—H3*A*⋯O2^i^	0.86	2.63	3.196 (3)	124
N3—H3*B*⋯N4^ii^	0.86	2.22	3.067 (3)	169
C19—H19⋯O2^iii^	0.93	2.54	3.294 (4)	139

Symmetry codes: (i) 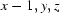; (ii) 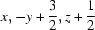; (iii) 
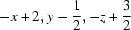
.

**Fig. 1.**
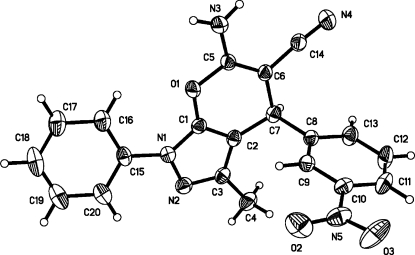
The molecular structure of the title compound, with atom labels and 50% probability displacement ellipsoids for non-H atoms.

**Fig. 2.**
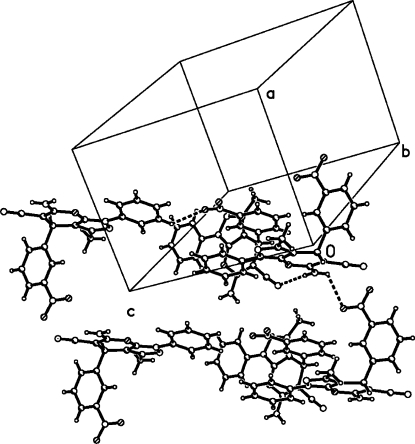
Crystal packing of the title compound, viewed along the a axis Intermolecular hydrogen bonds are shown as dashed lines.

## supplementary crystallographic information

### Comment

Multi-component reaction (MCR) protocols in water will be one of the most suitable strategies, which will meet the requirements of green chemistry as well as for developing libraries of medicinal scaffolds (Chanda *et al.*, 2009; Tejedor *et al.*, 2007). pyranopyrazoles are an important class of heterocyclic compounds. They found applications as pharmaceutical ingredients and biodegradable agrochemicals (Junek *et al.*, 1973; Wamhoff *et al.*, 1993; Sharanin *et al.*, 1983; Vasuki *et al.*, 2008). In order to further study the structure-activity relationship of pyranopyrazoles, we performed the synthesis of the title compound through an efficient and eco-friendly four-component one-pot reaction protocol in aqueous medium in the presence of catalytic amount dodecyltrimethylammonium bromide and present the crystal structure of the title compound in the hope that its structural features will appear interesting and helpful its practical applications. In the title molecule (Fig. 1), The pyranopyrazole ring essentially planar with a maximum deviation of 0.026 (0) Å from the least-squares plane defined by the nine constituent atoms. The dihedral angles formed by the mean plane of the pyranopyrazole fragment with the 3-nitrophenyl ring and phenyl ring is 81.11 (5)° and 13.36 (1)° , respectively. In the crystal the molecular packing (Fig. 2) is stabilized by Infinite chains via N-H···N hydrogen bonds propagate along c-axis, infinite chains via N-H···O hydrogen bonds propagate along a-axis and along a-axis there exists N-O···N-C dipole-dipole interactions with O···N distance of 3.061 Å which shorter than that of N-H···O (3.196 Å) hydrogen bond.

### Experimental

To a stirred aqueous mixture of phenylhydrazine ( 0.216g, 2 mmol) and ethyl acetoacetate ( 0.260g, 2 mmol), 3-nitrobenzaldehyde ( 0.302g, 2 mmol), malonitrile ( 0.132g, 2 mmol) and piperidine (5 mol %) were added successively at 363 K in the presence of catalytic amount dodecyltrimethylammonium bromide with vigorous stirring for 10 min. The precipitated solid was filtered, washed with water and then with a mixture of ethyl acetate/hexane (20:80). The product obtained was purified by flash chromatograghy. Single crystals of the title compound suitable for single-crystal X-ray analysis were obtained by recrystallization from ethanol.

### Refinement

H atoms bonded to N atoms were located in a difference map and refined with distance restraints of N—H = 0.860 Å, and with*U*_iso_(H) =1.2*U*_eq_(N). The remaining H atoms were positioned geometrically and refined using a riding model with C-H = 0.93-0.98 Å , and with *U*_iso_(H)= 1.2*U*_eq_(C).

### Crystal data

**Table:**

C_20_H_15_N_5_O_3_	*F*(000) = 776
*M**_r_* = 373.37	*D*_x_ = 1.410 Mg m^−^^3^
Monoclinic, *P*2_1_/*c*	Mo *K*α radiation, λ = 0.71073 Å
*a* = 9.5089 (8) Å	Cell parameters from 2416 reflections
*b* = 13.9137 (11) Å	θ = 2.6–23.5°
*c* = 13.3747 (12) Å	µ = 0.10 mm^−^^1^
β = 96.263 (1)°	*T* = 298 K
*V* = 1759.0 (3) Å^3^	Plate, colourless
*Z* = 4	0.50 × 0.48 × 0.47 mm

### Data collection

**Table:**

Bruker SMART CCD area-detector diffractometer	3087 independent reflections
Radiation source: fine-focus sealed tube	1961 reflections with *I* > 2σ(*I*)
graphite	*R*_int_ = 0.037
φ and ω scans	θ_max_ = 25.0°, θ_min_ = 2.6°
Absorption correction: multi-scan (*SADABS*; Bruker, 2002)	*h* = −11→11
*T*_min_ = 0.952, *T*_max_ = 0.955	*k* = −16→16
8659 measured reflections	*l* = −9→15

### Refinement

**Table:**

Refinement on *F*^2^	Secondary atom site location: difference Fourier map
Least-squares matrix: full	Hydrogen site location: inferred from neighbouring sites
*R*[*F*^2^ > 2σ(*F*^2^)] = 0.042	H-atom parameters constrained
*wR*(*F*^2^) = 0.121	*w* = 1/[σ^2^(*F*_o_^2^) + (0.0397*P*)^2^ + 0.6842*P*] where *P* = (*F*_o_^2^ + 2*F*_c_^2^)/3
*S* = 1.07	(Δ/σ)_max_ < 0.001
3087 reflections	Δρ_max_ = 0.19 e Å^−^^3^
255 parameters	Δρ_min_ = −0.17 e Å^−^^3^
0 restraints	Extinction correction: *SHELXL97* (Sheldrick, 2008), Fc^*^=kFc[1+0.001xFc^2^λ^3^/sin(2θ)]^-1/4^
Primary atom site location: structure-invariant direct methods	Extinction coefficient: 0.0100 (13)

### Special details

**Table:**

Geometry. All esds (except the esd in the dihedral angle between two l.s. planes) are estimated using the full covariance matrix. The cell esds are taken into account individually in the estimation of esds in distances, angles and torsion angles; correlations between esds in cell parameters are only used when they are defined by crystal symmetry. An approximate (isotropic) treatment of cell esds is used for estimating esds involving l.s. planes.
Refinement. Refinement of F^2^ against ALL reflections. The weighted R-factor wR and goodness of fit S are based on F^2^, conventional R-factors R are based on F, with F set to zero for negative F^2^. The threshold expression of F^2^ > 2sigma(F^2^) is used only for calculating R-factors(gt) etc. and is not relevant to the choice of reflections for refinement. R-factors based on F^2^ are statistically about twice as large as those based on F, and R- factors based on ALL data will be even larger.

### Fractional atomic coordinates and isotropic or equivalent isotropic displacement parameters (Å^2^)

**Table:**

	*x*	*y*	*z*	*U*_iso_*/*U*_eq_	
N1	0.71933 (19)	0.52908 (13)	0.63123 (13)	0.0440 (5)	
N2	0.8060 (2)	0.46605 (13)	0.58687 (15)	0.0507 (5)	
N3	0.3851 (2)	0.75499 (15)	0.52319 (15)	0.0565 (6)	
H3A	0.3339	0.7879	0.4785	0.068*	
H3B	0.3776	0.7641	0.5860	0.068*	
N4	0.3550 (2)	0.74422 (18)	0.24916 (17)	0.0741 (7)	
N5	1.0378 (2)	0.80263 (17)	0.3702 (2)	0.0674 (7)	
O1	0.54812 (16)	0.64832 (11)	0.57873 (10)	0.0471 (4)	
O2	1.0555 (2)	0.79273 (15)	0.46155 (19)	0.0900 (7)	
O3	1.1104 (2)	0.85581 (18)	0.3254 (2)	0.1121 (9)	
C1	0.6433 (2)	0.57976 (15)	0.55812 (16)	0.0404 (5)	
C2	0.6769 (2)	0.55266 (16)	0.46700 (16)	0.0418 (5)	
C3	0.7801 (2)	0.48064 (16)	0.48860 (18)	0.0466 (6)	
C4	0.8571 (3)	0.42635 (18)	0.4160 (2)	0.0625 (7)	
H4A	0.9190	0.4691	0.3853	0.094*	
H4B	0.7903	0.3987	0.3651	0.094*	
H4C	0.9118	0.3761	0.4508	0.094*	
C5	0.4761 (2)	0.68993 (16)	0.49508 (17)	0.0429 (6)	
C6	0.5006 (2)	0.66700 (16)	0.40010 (16)	0.0429 (6)	
C7	0.6133 (2)	0.59767 (16)	0.37153 (16)	0.0434 (6)	
H7	0.5673	0.5474	0.3283	0.052*	
C8	0.7221 (2)	0.64836 (16)	0.31459 (16)	0.0412 (5)	
C9	0.8293 (2)	0.70132 (16)	0.36589 (17)	0.0445 (6)	
H9	0.8368	0.7053	0.4357	0.053*	
C10	0.9247 (2)	0.74805 (16)	0.31350 (19)	0.0482 (6)	
C11	0.9187 (3)	0.74412 (19)	0.2108 (2)	0.0650 (7)	
H11	0.9848	0.7762	0.1767	0.078*	
C12	0.8126 (3)	0.6916 (2)	0.1602 (2)	0.0722 (8)	
H12	0.8063	0.6875	0.0904	0.087*	
C13	0.7148 (3)	0.64464 (19)	0.21116 (17)	0.0567 (7)	
H13	0.6426	0.6098	0.1752	0.068*	
C14	0.4173 (3)	0.71120 (18)	0.31880 (18)	0.0510 (6)	
C15	0.7217 (3)	0.52889 (16)	0.73764 (17)	0.0466 (6)	
C16	0.6181 (3)	0.57274 (18)	0.78491 (18)	0.0575 (7)	
H16	0.5454	0.6060	0.7476	0.069*	
C17	0.6223 (4)	0.5672 (2)	0.8885 (2)	0.0740 (9)	
H17	0.5521	0.5967	0.9208	0.089*	
C18	0.7290 (4)	0.5184 (2)	0.9436 (2)	0.0851 (10)	
H18	0.7303	0.5137	1.0131	0.102*	
C19	0.8331 (4)	0.4768 (2)	0.8965 (2)	0.0856 (10)	
H19	0.9066	0.4449	0.9343	0.103*	
C20	0.8312 (3)	0.4813 (2)	0.7935 (2)	0.0684 (8)	
H20	0.9028	0.4527	0.7619	0.082*	

### Atomic displacement parameters (Å^2^)

**Table:**

	*U*^11^	*U*^22^	*U*^33^	*U*^12^	*U*^13^	*U*^23^
N1	0.0465 (11)	0.0447 (11)	0.0404 (11)	0.0003 (9)	0.0034 (9)	0.0016 (9)
N2	0.0507 (12)	0.0471 (12)	0.0553 (13)	0.0015 (10)	0.0105 (10)	0.0039 (10)
N3	0.0560 (12)	0.0692 (14)	0.0443 (12)	0.0159 (11)	0.0054 (10)	0.0013 (10)
N4	0.0683 (15)	0.1012 (19)	0.0511 (14)	0.0056 (14)	−0.0008 (12)	0.0171 (14)
N5	0.0482 (14)	0.0591 (14)	0.096 (2)	−0.0073 (11)	0.0149 (14)	−0.0085 (15)
O1	0.0511 (9)	0.0555 (10)	0.0340 (8)	0.0053 (8)	0.0018 (7)	−0.0017 (7)
O2	0.0833 (15)	0.0804 (14)	0.0994 (18)	−0.0252 (12)	−0.0219 (14)	−0.0020 (14)
O3	0.0880 (16)	0.1111 (18)	0.147 (2)	−0.0534 (15)	0.0570 (16)	−0.0233 (16)
C1	0.0384 (12)	0.0439 (13)	0.0394 (13)	−0.0043 (11)	0.0059 (10)	−0.0012 (11)
C2	0.0441 (13)	0.0418 (12)	0.0403 (13)	−0.0090 (10)	0.0079 (11)	0.0008 (10)
C3	0.0472 (14)	0.0436 (13)	0.0508 (15)	−0.0072 (11)	0.0140 (12)	0.0025 (11)
C4	0.0662 (17)	0.0579 (16)	0.0672 (17)	0.0054 (13)	0.0251 (14)	−0.0015 (14)
C5	0.0384 (12)	0.0509 (14)	0.0385 (13)	−0.0049 (11)	0.0008 (10)	0.0035 (11)
C6	0.0380 (12)	0.0541 (14)	0.0366 (13)	−0.0080 (11)	0.0035 (10)	0.0033 (11)
C7	0.0459 (13)	0.0486 (13)	0.0355 (13)	−0.0096 (11)	0.0043 (11)	−0.0063 (10)
C8	0.0429 (13)	0.0458 (13)	0.0351 (12)	0.0002 (10)	0.0056 (10)	−0.0018 (10)
C9	0.0458 (13)	0.0491 (13)	0.0390 (13)	0.0013 (11)	0.0061 (11)	−0.0019 (11)
C10	0.0412 (13)	0.0444 (13)	0.0606 (16)	−0.0014 (11)	0.0126 (12)	−0.0014 (12)
C11	0.0699 (18)	0.0646 (17)	0.0664 (18)	−0.0081 (15)	0.0342 (15)	0.0064 (15)
C12	0.089 (2)	0.090 (2)	0.0416 (15)	−0.0100 (18)	0.0238 (15)	0.0038 (15)
C13	0.0625 (16)	0.0697 (17)	0.0384 (14)	−0.0096 (14)	0.0074 (12)	−0.0054 (13)
C14	0.0469 (14)	0.0646 (16)	0.0416 (14)	−0.0040 (12)	0.0058 (12)	0.0037 (13)
C15	0.0578 (15)	0.0422 (13)	0.0382 (13)	−0.0122 (12)	−0.0012 (12)	0.0036 (11)
C16	0.0750 (18)	0.0536 (15)	0.0437 (15)	−0.0003 (13)	0.0049 (13)	−0.0018 (12)
C17	0.109 (2)	0.0685 (19)	0.0459 (16)	−0.0019 (17)	0.0144 (17)	−0.0063 (14)
C18	0.142 (3)	0.071 (2)	0.0397 (16)	−0.008 (2)	−0.005 (2)	0.0044 (15)
C19	0.113 (3)	0.081 (2)	0.056 (2)	0.008 (2)	−0.0179 (19)	0.0136 (17)
C20	0.0764 (19)	0.0700 (18)	0.0561 (17)	0.0042 (15)	−0.0050 (15)	0.0104 (14)

### Geometric parameters (Å, °)

**Table:**

N1—C1	1.350 (3)	C7—C8	1.523 (3)
N1—N2	1.381 (2)	C7—H7	0.9800
N1—C15	1.421 (3)	C8—C9	1.378 (3)
N2—C3	1.326 (3)	C8—C13	1.379 (3)
N3—C5	1.334 (3)	C9—C10	1.369 (3)
N3—H3A	0.8600	C9—H9	0.9300
N3—H3B	0.8600	C10—C11	1.370 (3)
N4—C14	1.144 (3)	C11—C12	1.364 (4)
N5—O3	1.214 (3)	C11—H11	0.9300
N5—O2	1.222 (3)	C12—C13	1.377 (4)
N5—C10	1.460 (3)	C12—H12	0.9300
O1—C1	1.364 (3)	C13—H13	0.9300
O1—C5	1.374 (2)	C15—C16	1.371 (3)
C1—C2	1.347 (3)	C15—C20	1.382 (3)
C2—C3	1.411 (3)	C16—C17	1.384 (3)
C2—C7	1.490 (3)	C16—H16	0.9300
C3—C4	1.484 (3)	C17—C18	1.367 (4)
C4—H4A	0.9600	C17—H17	0.9300
C4—H4B	0.9600	C18—C19	1.360 (4)
C4—H4C	0.9600	C18—H18	0.9300
C5—C6	1.354 (3)	C19—C20	1.377 (4)
C6—C14	1.414 (3)	C19—H19	0.9300
C6—C7	1.522 (3)	C20—H20	0.9300
			
C1—N1—N2	108.55 (17)	C9—C8—C13	118.2 (2)
C1—N1—C15	132.5 (2)	C9—C8—C7	120.29 (19)
N2—N1—C15	118.94 (18)	C13—C8—C7	121.5 (2)
C3—N2—N1	105.87 (18)	C10—C9—C8	119.6 (2)
C5—N3—H3A	120.0	C10—C9—H9	120.2
C5—N3—H3B	120.0	C8—C9—H9	120.2
H3A—N3—H3B	120.0	C9—C10—C11	122.5 (2)
O3—N5—O2	122.6 (3)	C9—C10—N5	118.2 (2)
O3—N5—C10	119.1 (3)	C11—C10—N5	119.3 (2)
O2—N5—C10	118.3 (2)	C12—C11—C10	117.8 (2)
C1—O1—C5	114.40 (17)	C12—C11—H11	121.1
C2—C1—N1	110.4 (2)	C10—C11—H11	121.1
C2—C1—O1	127.4 (2)	C11—C12—C13	120.8 (2)
N1—C1—O1	122.27 (19)	C11—C12—H12	119.6
C1—C2—C3	104.0 (2)	C13—C12—H12	119.6
C1—C2—C7	123.0 (2)	C12—C13—C8	121.1 (2)
C3—C2—C7	133.0 (2)	C12—C13—H13	119.5
N2—C3—C2	111.2 (2)	C8—C13—H13	119.5
N2—C3—C4	121.2 (2)	N4—C14—C6	175.8 (3)
C2—C3—C4	127.5 (2)	C16—C15—C20	120.0 (2)
C3—C4—H4A	109.5	C16—C15—N1	121.8 (2)
C3—C4—H4B	109.5	C20—C15—N1	118.1 (2)
H4A—C4—H4B	109.5	C15—C16—C17	119.5 (3)
C3—C4—H4C	109.5	C15—C16—H16	120.2
H4A—C4—H4C	109.5	C17—C16—H16	120.2
H4B—C4—H4C	109.5	C18—C17—C16	120.4 (3)
N3—C5—C6	127.4 (2)	C18—C17—H17	119.8
N3—C5—O1	109.72 (19)	C16—C17—H17	119.8
C6—C5—O1	122.9 (2)	C19—C18—C17	119.7 (3)
C5—C6—C14	118.6 (2)	C19—C18—H18	120.1
C5—C6—C7	125.7 (2)	C17—C18—H18	120.1
C14—C6—C7	115.70 (19)	C18—C19—C20	120.9 (3)
C2—C7—C6	106.39 (18)	C18—C19—H19	119.5
C2—C7—C8	112.92 (18)	C20—C19—H19	119.5
C6—C7—C8	111.56 (18)	C19—C20—C15	119.3 (3)
C2—C7—H7	108.6	C19—C20—H20	120.3
C6—C7—H7	108.6	C15—C20—H20	120.3
C8—C7—H7	108.6		
			
C1—N1—N2—C3	0.1 (2)	C2—C7—C8—C9	−40.3 (3)
C15—N1—N2—C3	178.88 (19)	C6—C7—C8—C9	79.5 (2)
N2—N1—C1—C2	−0.1 (2)	C2—C7—C8—C13	141.2 (2)
C15—N1—C1—C2	−178.6 (2)	C6—C7—C8—C13	−99.0 (2)
N2—N1—C1—O1	−179.75 (18)	C13—C8—C9—C10	−0.2 (3)
C15—N1—C1—O1	1.7 (3)	C7—C8—C9—C10	−178.7 (2)
C5—O1—C1—C2	3.0 (3)	C8—C9—C10—C11	−0.3 (4)
C5—O1—C1—N1	−177.44 (18)	C8—C9—C10—N5	−179.3 (2)
N1—C1—C2—C3	0.1 (2)	O3—N5—C10—C9	−169.2 (2)
O1—C1—C2—C3	179.7 (2)	O2—N5—C10—C9	10.8 (3)
N1—C1—C2—C7	−178.24 (19)	O3—N5—C10—C11	11.8 (4)
O1—C1—C2—C7	1.4 (3)	O2—N5—C10—C11	−168.2 (3)
N1—N2—C3—C2	−0.1 (2)	C9—C10—C11—C12	0.3 (4)
N1—N2—C3—C4	178.7 (2)	N5—C10—C11—C12	179.3 (2)
C1—C2—C3—N2	0.0 (3)	C10—C11—C12—C13	0.3 (4)
C7—C2—C3—N2	178.1 (2)	C11—C12—C13—C8	−0.7 (4)
C1—C2—C3—C4	−178.7 (2)	C9—C8—C13—C12	0.7 (4)
C7—C2—C3—C4	−0.7 (4)	C7—C8—C13—C12	179.2 (2)
C1—O1—C5—N3	179.41 (18)	C5—C6—C14—N4	175 (4)
C1—O1—C5—C6	−2.0 (3)	C7—C6—C14—N4	−5(4)
N3—C5—C6—C14	−4.0 (4)	C1—N1—C15—C16	13.3 (4)
O1—C5—C6—C14	177.69 (19)	N2—N1—C15—C16	−165.1 (2)
N3—C5—C6—C7	175.1 (2)	C1—N1—C15—C20	−167.8 (2)
O1—C5—C6—C7	−3.3 (3)	N2—N1—C15—C20	13.8 (3)
C1—C2—C7—C6	−5.6 (3)	C20—C15—C16—C17	−1.4 (4)
C3—C2—C7—C6	176.6 (2)	N1—C15—C16—C17	177.4 (2)
C1—C2—C7—C8	117.1 (2)	C15—C16—C17—C18	0.1 (4)
C3—C2—C7—C8	−60.7 (3)	C16—C17—C18—C19	1.3 (5)
C5—C6—C7—C2	6.7 (3)	C17—C18—C19—C20	−1.4 (5)
C14—C6—C7—C2	−174.27 (19)	C18—C19—C20—C15	0.0 (5)
C5—C6—C7—C8	−116.9 (2)	C16—C15—C20—C19	1.4 (4)
C14—C6—C7—C8	62.2 (2)	N1—C15—C20—C19	−177.5 (2)

### Hydrogen-bond geometry (Å, °)

**Table:**

*D*—H···*A*	*D*—H	H···*A*	*D*···*A*	*D*—H···*A*
N3—H3A···O2^i^	0.86	2.63	3.196 (3)	124
N3—H3B···N4^ii^	0.86	2.22	3.067 (3)	169
C19—H19···O2^iii^	0.93	2.54	3.294 (4)	139

Symmetry codes: (i) *x*−1, *y*, *z*; (ii) *x*, −*y*+3/2, *z*+1/2; (iii) −*x*+2, *y*−1/2, −*z*+3/2.
